# Supplementary material for: Using instrumental variables to disentangle treatment and placebo effects in blinded and unblinded randomized clinical trials influenced by unmeasured confounders
Source: Sci Rep. 2016 Nov 21;6:37154. doi: 10.1038/srep37154 (PMC5116680; doi:10.1038/srep37154)
Supplement: Supplementary Information [file srep37154-s1.pdf]

Supplementary Information for: Using instrumental variables to disentangle treatment and placebo effects in blinded and unblinded randomized clinical trials influenced by unmeasured confounders

by Elias Chaibub Neto

Supplementary Figures

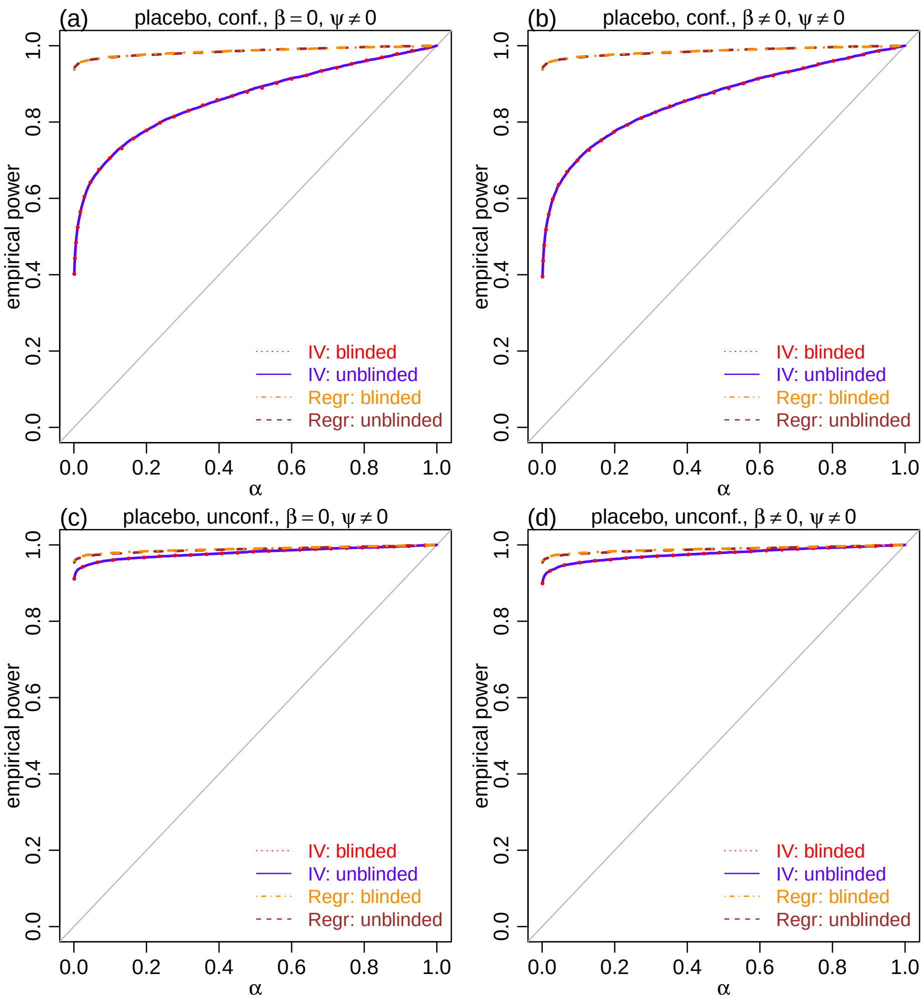

Supplementary Figure 1. Empirical power to detect placebo effects in the blinded and unblinded settings. Panels a and b show the results in the presence of confounders, whereas panels c and d show the results in their absence. The regression approach (brown and dark-orange) were considerably better powered than the IV approaches (blue and red) in the presence of confounders (panels a and b), but only slightly better powered in the absence of confounders (panels c and d). Both regression and IV approaches showed similar power under the blinded and unblinded settings.

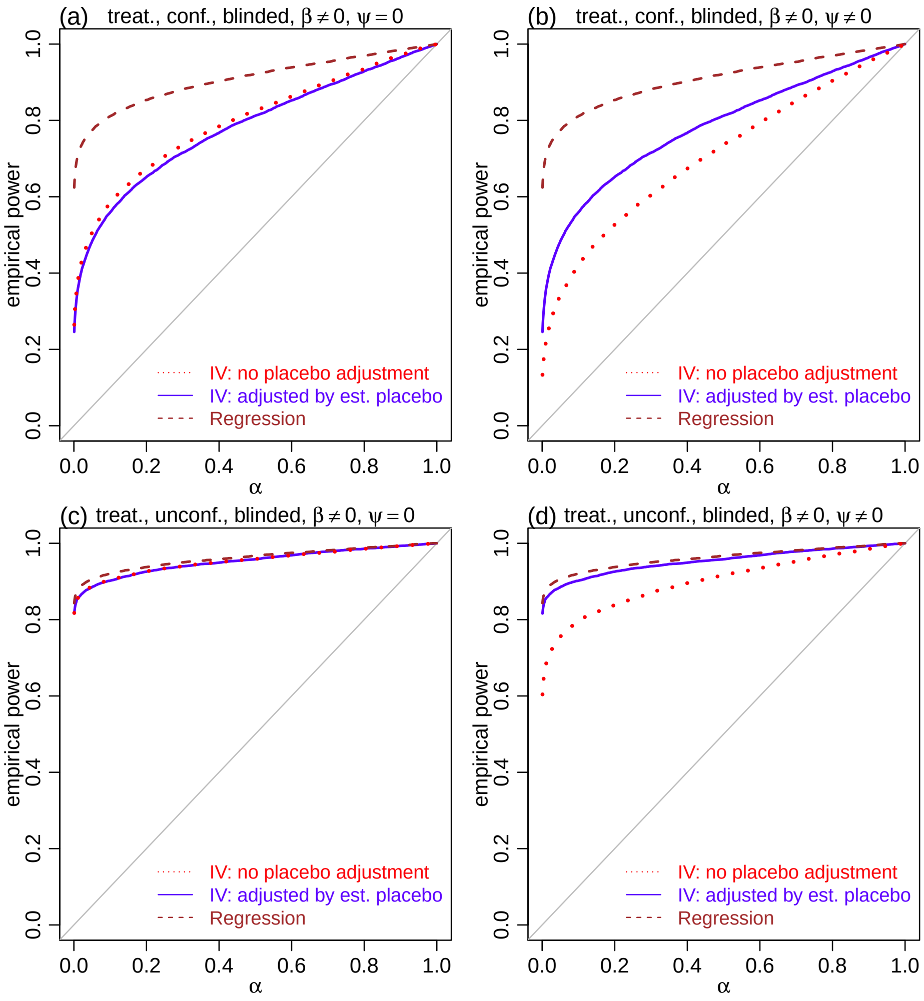

Supplementary Figure 2. Empirical power for detecting treatment effects in the blinded setting. The regression approach (brown) tends to be better powered than the IV approaches in the presence of confounders (panels a and b), but only slightly better in the absence of confounding (panels c and d). The two-step IV approach (blue) tends to be better powered than the non-adjusted one (red) in the presence of placebo effects (panels b and d), but both IV approaches tend to be comparable in absence of placebo effects (panels a and c).

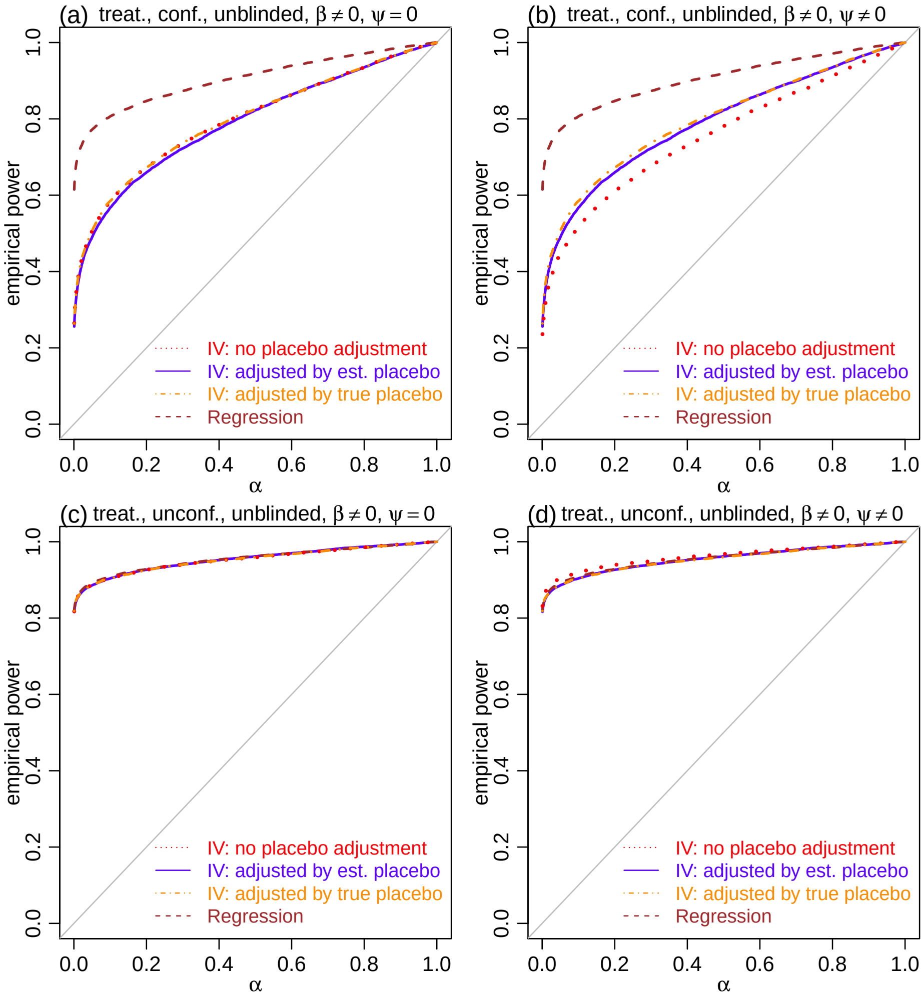

Supplementary Figure 3. Empirical power for detecting treatment effects in the unblinded setting. The regression approach (brown) tended to be better powered than the IV approaches in the presence of confounders (panels a and b), but comparable in the absence of confounding (panels c and d). The two-step IV approach (blue) tended to be slightly better powered than the non-adjusted one (red) in the presence of placebo effect (panel b), but comparable in the other panels.

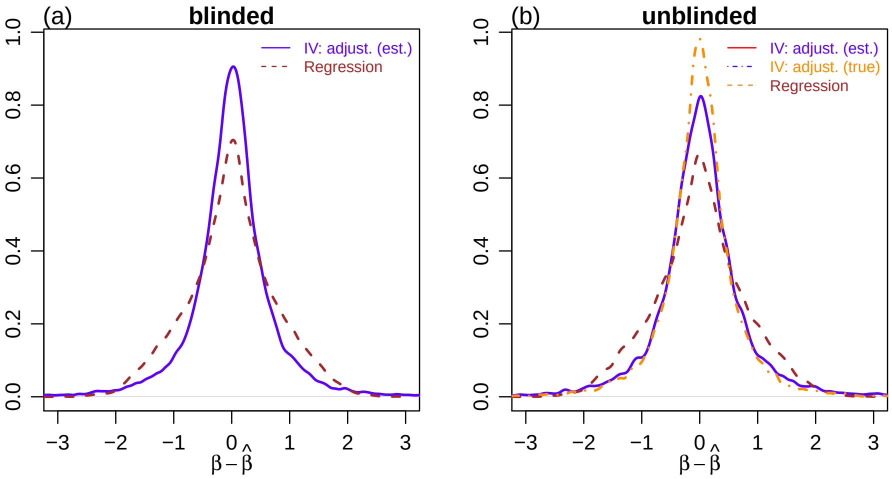

Supplementary Figure 4. Comparison of the bias of the regression and IV estimators. Panels a and b show the densities of the difference between true and estimated treatment effects, $\beta - \hat{\beta}$, in the blinded and unblinded settings, respectively. In both settings we observed larger bias in the regression estimates, in comparison to the IV approaches, as illustrated by the heavier tails of the brown densities.

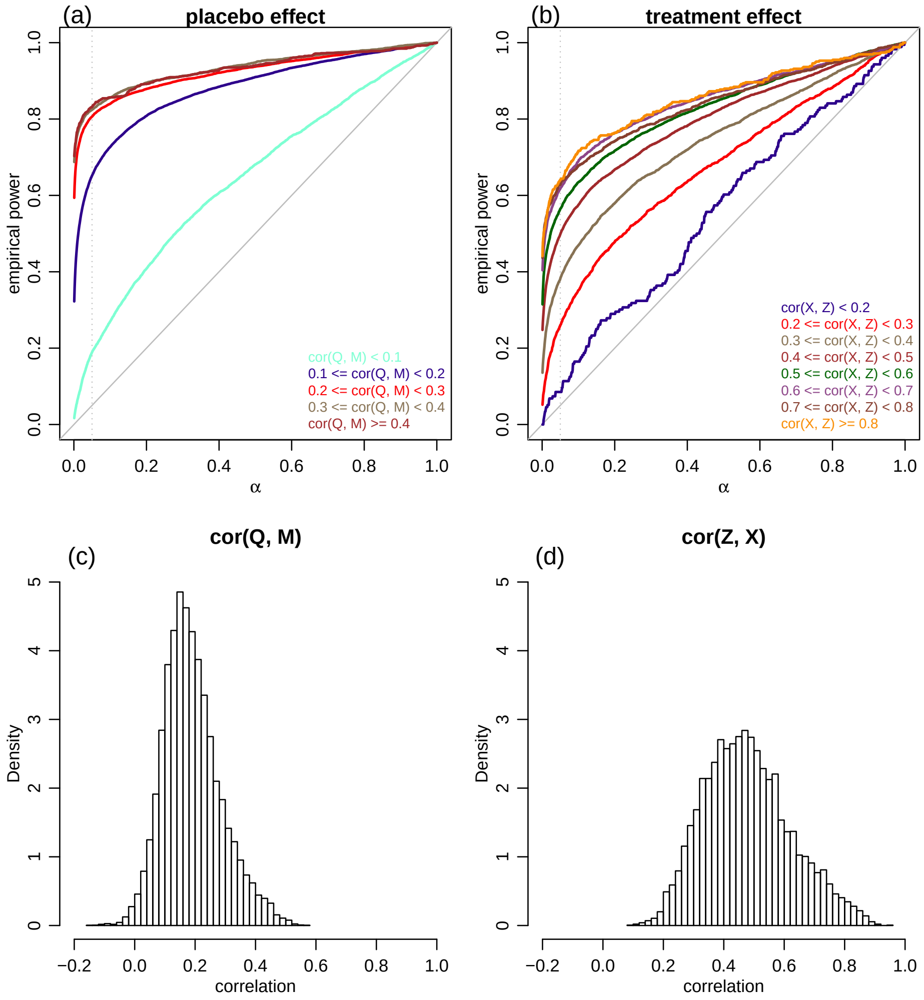

Supplementary Figure 5. Empirical power curves stratified by strength of association with the IV variable. Panel a shows the power curves for the placebo effect IV estimator $\hat{\psi}$, stratified according to the correlation between Q and M (panel c shows the distribution of the correlation between Q and M across all simulations used to construct the power curves in panel a). Panel b shows the power curves for the two-step treatment effect IV estimator $\hat{\beta}$, stratified according to the correlation between Z and X (panel d shows the distribution of the correlation between Z and X over the simulations used to estimate the power curves in panel b). Results based on blinded and unblinded simulations influence by confounders.

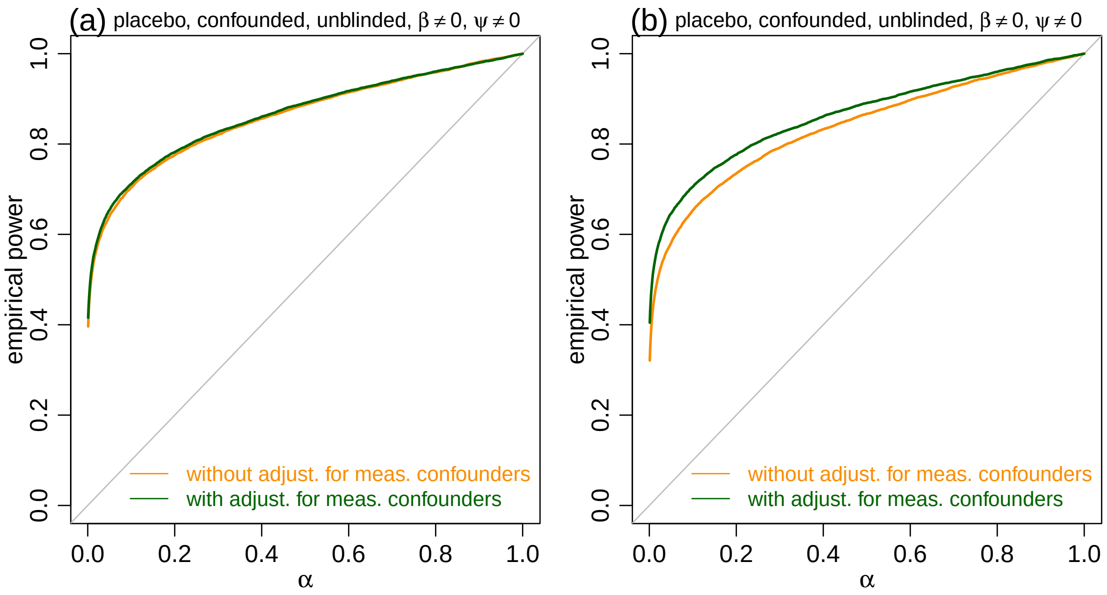

Supplementary Figure 6. Empirical power comparison of the IV estimator of placebo effects, with and without adjustment for measured confounders. In order to illustrate how the adjustment for measured confounders can improve the power to detect a causal effect, we show how the use of the treatment variable as a measured confounder of the placebo effect can improve the power to detect placebo effects. Note that in unblinded trials, X corresponds to a measured confounder of the placebo effect ψ, since it influences both M (via E) and Y, as illustrated in Figure 1 in the main text. Panel a shows the comparison for data simulated under the unblinded and confounded setting in the presence of treatment and placebo effects, as described in the main text. In this case, adjustment for X seems to provide a marginal improvement in the power to detect placebo effects. Panel b shows the comparison for data simulated under the same specifications as in panel a, but using stronger effects of X on Y and of X on M (via stronger effects of X on E, and of E on M). In this situation, adjustment for X does improve the power to detect placebo effects by a considerably larger margin.

Supplementary Note

Performance evaluation when the emotion level variable is influenced by measurement error

In the following additional simulation studies, involving measurement error, we focus only on the unblinded/unconfounded and on the unblinded/confounded simulation settings in the presence of both placebo and treatment effects (i.e., $\beta \neq 0$ and $\psi \neq 0$). For each simulated data set we first generate data for the (Z, X, Q, M, Y) variables (in exactly the same manner as the respective simulations presented in the main text), but then generate a new emotion level variable, $\tilde{M}$, by introducing measurement error (ME) on the original emotion level variable, M, according to the linear model, $\tilde{M} = M + \varepsilon_{ME}$, where $\varepsilon_{ME} \sim N(0, \sigma^2_{ME})$. (Note that we still use the perfectly measurement value, M, in the generation of Y, but run our analysis using the $(Z, X, Q, \tilde{M}, Y)$ data.) We consider three distinct levels of measurement error, the "moderate ME" setting, where $\sigma^2_{ME} = 5$, the "high ME" setting, with $\sigma^2_{ME} = 25$, and the "extreme ME" setting, where $\sigma^2_{ME} = 100$. Figures 1 and 2 illustrate that these particular choices indeed represent moderate, high, and extreme levels of ME, by comparing the variance of the emotion levels in the original data (no ME) to the variance of the emotion levels generated under the influence of these amounts of ME. In total, we considered 6 additional simulation experiments, each one encompassing 10,000 distinct data sets.

Panels a, b, and c of Figure 3 present, respectively, the empirical bias, $\psi - \hat{\psi}$, of the placebo effect estimator for moderate, high, and extreme levels of ME, for data simulated in the absence of confounders. The panels clearly show that the IV estimator (blue) produces considerably less biased placebo effect estimates than the regression estimator (brown). This result is not surprising given that the IV estimator is able to handle measurement error in explanatory variables (as a matter of fact, it has been pointed out[1] that the original motivation for the development of IV methods in the econometrics field was to account for measurement error in the explanatory variables, and that only later IVs have been used to account for unmeasured confounders). To see how the placebo effect IV estimator is able to account for ME in the explanatory variable M observe that (at least for reasonably large sample sizes),

$$\hat{\psi}_{IV} = \frac{\widehat{\text{Cov}}(Q, Y)}{\widehat{\text{Cov}}(Q, M)} \approx \frac{\widehat{\text{Cov}}(Q, Y)}{\widehat{\text{Cov}}(Q, \tilde{M})} , \tag{1}$$

since $\widehat{\text{Cov}}(Q, M)$ and $\widehat{\text{Cov}}(Q, \tilde{M})$ are consistent estimators of $\text{Cov}(Q, M)$ and $\text{Cov}(Q, \tilde{M})$ and,

$$\text{Cov}(Q, \tilde{M}) = \text{Cov}(Q\,, M + \varepsilon_{ME}) = \text{Cov}(Q, M) + \text{Cov}(Q, \varepsilon_{ME}) = \text{Cov}(Q, M) \ , \tag{2}$$

where the last equality follows from the fact that $\text{Cov}(Q, \varepsilon_{ME}) = 0$ (since Q is randomized), so that $\widehat{\text{Cov}}(Q, M) \approx \widehat{\text{Cov}}(Q, \tilde{M})$ in reasonably large sample sizes.

Panels d, e, and f of Figure 3 present the distributions of the placebo effects estimates generated by the IV (blue) and regression (brown) estimators along with the distributions of the true placebo effect values (black) used in the simulation of the synthetic data. These panels clarify that the peculiar shape of the brown densities in the top panels is explained by the fact that the placebo effect estimates generated by regression tend to get more concentrated around zero, as the amount of measurement error increases, so that the bias distribution ends up closely approximating the distribution of ψ (compare the brown density in panel c to the black density in panel d of Figure 3). On the other hand, the distributions of the estimates generated by the IV estimator tend to be much closer to the distribution of the true placebo effects (note the similarity between the blue and black densities). Figure 3, also show that the amount of bias tends to increase with increasing amounts of ME, as one would expect.

Figure 4 presents the results for data simulated in the presence of confounders. We observe essentially the same patterns, except that the results are not as clear cut as in the unconfounded case (note the different scales in the y-axis in comparison to Figure 3). This observation is not surprising given that the presence of confounding makes statistical inferences more challenging.

Figure 5 presents the empirical power curves for the placebo effect estimators for both the unconfounded (panel a) and confounded (panel b) cases. Panel a shows that in the absence of confounding, the IV estimators tended to show high empirical power independent of the amount of ME used in the simulations, whereas the power of the regression approach tended to decrease with increasing amounts of ME. Panel b, on the other hand, show that the IV estimator tended to be less powered than the regression one in the presence of confounding. We point out, however, that the high empirical power achieved by the regression estimator in the presence of confounding seems to be an artifact of the highly biased estimates produced by the regression approach, as illustrated in the top panels of Figure 4.

It is interesting to note that the randomization test based on the placebo effect IV estimator is robust to measurement error, as illustrated by the fact that the power curves for the distinct amounts of ME (full, dashed, and dotted blue lines in Figure 5) lay on top of each other. To see why this is the case, recall that in the generation of the randomization null we only shuffle the

response data, Y, relative to the the instrument and emotion level data, (Q,M), whose association is kept intact. Hence, while the numerator of the IV estimator, $\widehat{\text{Cov}}(Q,Y)$, changes with distinct data shufflyings, the denominator, $\widehat{\text{Cov}}(Q,M)$, is constant across all shufflyings used to generate the randomization null. Therefore, it follows that even though the randomization null distributions based on the statistics,

$$\frac{\widehat{\text{Cov}}(Q,Y)}{\widehat{\text{Cov}}(Q,M)}\,, \qquad \frac{\widehat{\text{Cov}}(Q,Y)}{\widehat{\text{Cov}}(Q,\tilde{M})}\,, \tag{3}$$

have different spreads (since the distinct but constant denominators scale the identical numerators differently), they still represent a simple re-scaling of each other, and generate the exact same p-values (as long as we use the same permutations of the response data in the generation of the randomization null distributions). Hence, the randomization test generates the same conclusion for an analysis based on perfectly measured emotion levels as in an analysis based on emotion data affected by an arbitrary amount of measurement error. Figure 6 shows an illustrative example.

Figure 7 presents the distributions of the treatment effect bias, $\beta - \hat{\beta}$ (top panels), and treatment effect estimates (bottom panels) for data simulated under the influence of moderate, high, and extreme levels of ME, in the absence of confounding. In addition to the regression estimator, $\hat{\beta}_R$ (brown), and placebo adjusted IV estimator, $\hat{\beta}_{2sIV} = \widehat{\text{Cov}}(Z,\hat{R})/\widehat{\text{Cov}}(Z,X)$ (blue), the figure also report results for the unadjusted IV estimator, $\hat{\beta}_{IV} = \widehat{\text{Cov}}(Z,Y)/\widehat{\text{Cov}}(Z,X)$ (red). The simulation results show that the $\hat{\beta}_{2sIV}$ estimator tended to outperform $\hat{\beta}_{IV}$ and $\hat{\beta}_R$ in the presence of measurement error, although the decreases in bias achieved by $\hat{\beta}_{2sIV}$ tended to be less accentuated in comparison to the decreases observed for the placebo effect estimator (note the different scales in the y-axis of Figure 7 in comparison to Figure 3). This observation is also not surprising since the estimation of ψ is never free from noise, and, even though $\hat{\psi}_{IV}$ is able to reduce the additional bias induced by ME, it cannot completely neutralize it. Hence, the placebo effect estimates obtained in the presence of ME, and employed in the computation of the residuals, $\widehat{R} = Y - \hat{\psi}_{IV}\,\tilde{M}$ (used in the estimation of the $\hat{\beta}_{2sIV}$), tend to be less effective in removing the influence of the placebo effect on the outcome variable.

Figure 8 presents the respective results in the presence of confounding. It shows that the presence of both confounding and measurement error makes the statistical inference considerably more challenging. Panel a shows that the $\hat{\beta}_{2sIV}$ etimator (blue) produces less biased estimates than $\hat{\beta}_{IV}$ (red) and $\hat{\beta}_R$ (brown) in the presence of moderate amounts of measurement error. Panel b shows that the bias density of the $\hat{\beta}_{2sIV}$ estimator is slightly more peaked around 0, but also have heavier tails than the densities of the $\hat{\beta}_{IV}$ (red) and $\hat{\beta}_R$ (brown) estimators. Panel c, however, clearly shows that the $\hat{\beta}_{2sIV}$ estimator can be more biased than the other estimators in the presence of confounding and extreme amounts of measurement error (note how the blue density puts less mass around 0, and have considerably heavier tails than the red and brown densities). The likely reason is that, in the presence of extreme amounts of ME, the $\hat{\psi}_{IV}$ estimator can still be fairly biased (as illustrated in panel c of Figure 4), so that adjustments based on a highly biased estimates of ψ end up harming the estimation of the treatment effect. We point out, nonetheless, that our evaluations included such extreme amounts of ME for illustrative purposes (since we wanted to investigate how much ME $\hat{\beta}_{2sIV}$ would be able to handle, before it started performing worse than $\hat{\beta}_{IV}$). However, in reality, such extreme cases are not very likely to be found in practice. (Recall that the emotion level variable was, on average, 14 times more variable than the original data free from ME in the extreme ME setting).

Figure 9 presents the empirical power curves for the treatment effect estimators for both the unconfounded (panel a) and confounded (panel b) cases. In both panels the regression and unadjusted IV estimators tended to be higher powered than the placebo adjusted IV estimator (whose power tended to decrease with increasing amounts of ME).

Finally, for the sake of completeness, we also evaluate the type I error rates of the procedures in the presence of ME, using a few additional simulation experiments focusing again in the unblinded/unconfounded and on the unblinded/confounded settings, but in the absence of placebo and treatment effects (i.e., $\beta = 0$ and $\psi = 0$). Panels a and b of Figure 10 present the results for the placebo effect tests in the unconfounded and confounded cases, respectively, and show that the error rates of the IV approach (blue) are still controlled at the exact nominal levels in the presence of measurement error, whereas the regression approach (brown) still shows inflated error rates in the presence of confounding. Panels c and d present the respective results for the treatment effect tests. Both panels show well controlled error rates for the unadjusted IV approach (red), but slightly inflated error rates for the placebo adjusted IV approach (blue). The regression approach (brown), on the other hand, shows well controlled error rates in the absence of confounding (panel c) but inflated errors in the presence of confounding (panel d).

References

1. Angrist, J. & Krueger, A. Instrumental variables and the search for identification: from supply and demand to natural experiments. *Journal of Economic Perspectives*, **15**, 69-85 (2001).

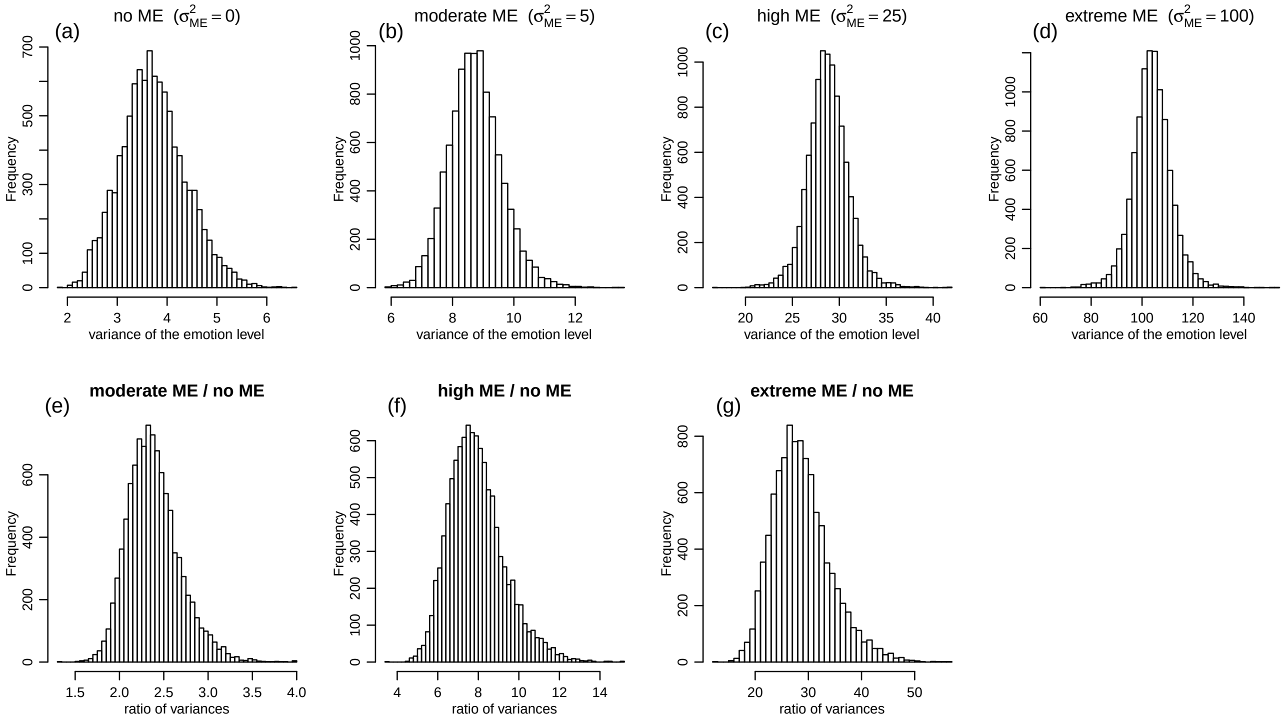

Figure 1. Comparison of the emotion level variances under varying levels of measurement error in the absence of confounding. Panel a shows the distribution of estimates of the variance of the emotion level across 10,000 synthetic data sets simulated (without measurement error) under the unblinded/unconfounded setting, in the presence of treatment and placebo effects. Panels b, c, and d show the distributions of the variance estimates after the addition of moderate, high and extreme amounts of measurement error to the original data, respectively. Panel e shows the distribution of the ratio of the variances for data generated under moderate ME relative to the original data. Note that the mean is around 2.4, meaning that, on average, the data generated with ME was 2.4 times more variable than the original data, showing that the choice $\sigma^2_{ME} = 5$ induces a moderate amount of ME in the data. Panel f shows the respective distribution for data generated under high ME relative to the original data. In this case, the data generated with measurement error was on average 8 times more variable than the original data, showing that the choice $\sigma^2_{ME} = 25$ indeed leads to high ME levels. Panel g shows the distribution of the ratio of the variances for the comparison of extreme ME relative to the original data. On average, the data generated with ME was 29 times more variable than the original data, showing that the choice $\sigma^2_{ME} = 100$ leads to extreme ME levels in the data.

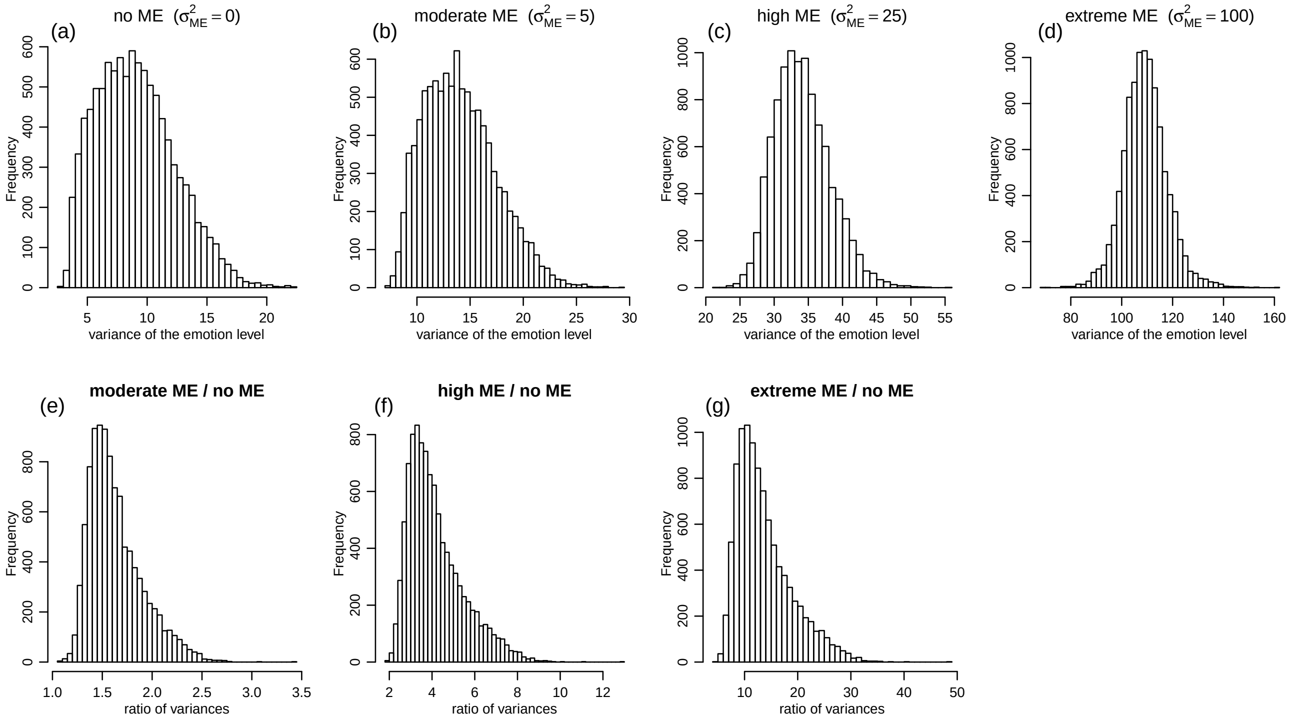

Figure 2. Comparison of the emotion level variances under varying levels of measurement error in the presence of confounding. Panel a shows the distribution of estimates of the variance of the emotion level across 10,000 synthetic data sets simulated (without measurement error) under the unblinded/confounded setting, in the presence of treatment and placebo effects. Panels b, c, and d show the distributions of the variance estimates after the addition of moderate, high and extreme amounts of measurement error to the original data, respectively. Panel e shows the distribution of the ratio of the variances for data generated under moderate ME relative to the original data. Note that the mean is around 1.6, meaning that, on average, the data generated with ME was 1.6 times more variable than the original data, showing that the choice $\sigma^2_{ME} = 5$ induces a moderate amount of ME in the data. Panel f shows the respective distribution for data generated under high ME relative to the original data. In this case, the data generated with measurement error was on average 4 times more variable than the original data, showing that the choice $\sigma^2_{ME} = 25$ indeed leads to high ME levels. Panel g shows the distribution of the ratio of the variances for the comparison of extreme ME relative to the original data. On average, the data generated with ME was 14 times more variable than the original data, showing that the choice $\sigma^2_{ME} = 100$ leads to extreme ME levels in the data.

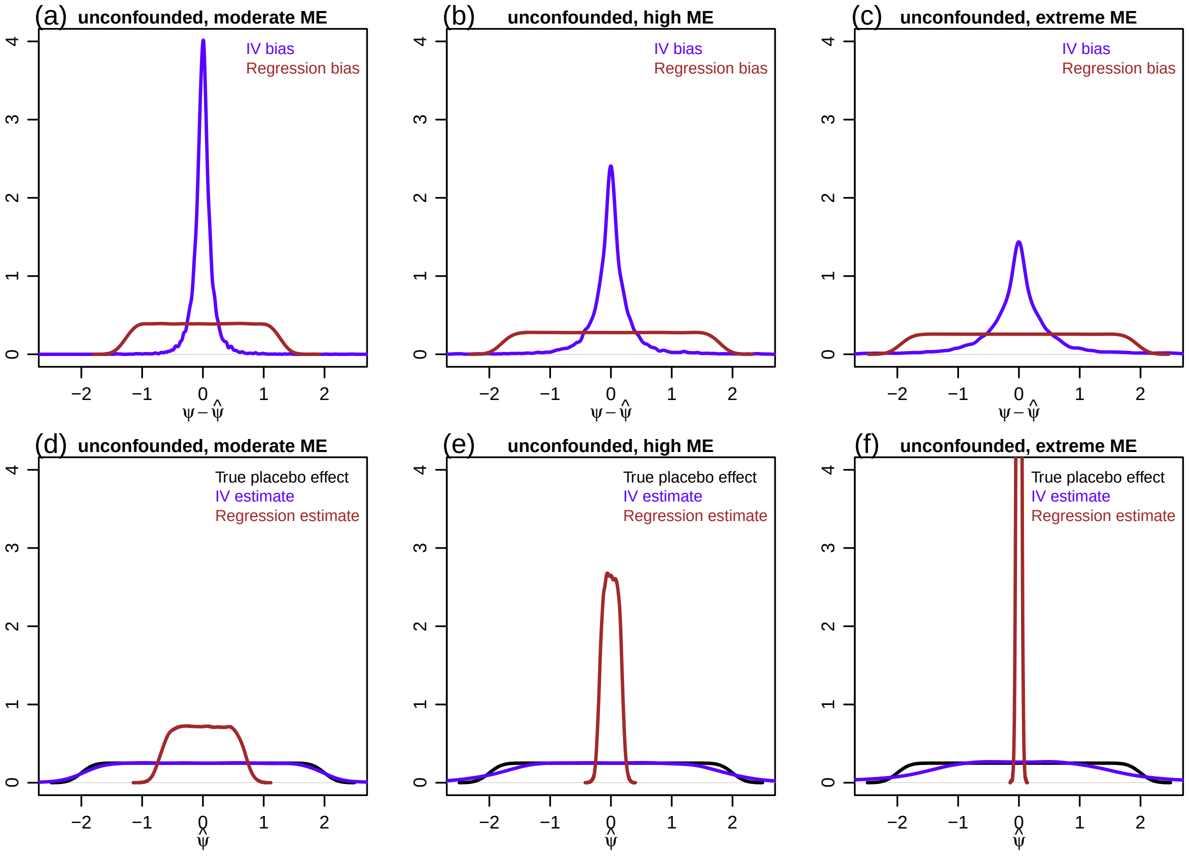

Figure 3. Empirical bias of the placebo effect tests under the influence of measurement error and in the absence of confounding. Panels a, b, and c show density estimates of the placebo effect bias, $\psi - \hat{\psi}$, for the IV (blue) and regression (brown) estimators, for the data sets simulated under the influence of moderate, high, and extreme amounts of measurement error of the emotion level variable, but in the absence of confounding. The panels clearly show that the IV estimator (blue) produces considerably less biased placebo effect estimates than the regression estimator (brown), and that the amount of bias tends to increase with increasing amounts of measurement error (as one would expect). Panels d, e, and f present the distributions of the placebo effects estimates generated by the IV (blue) and regression (brown) estimators along with the distributions of the true placebo effects (black) used to simulate the synthetic data. These panels show that the placebo effect estimates generated by the regression approach tend to get more concentrated around zero, as the amount of measurement error increases, whereas the estimates generated by the IV estimator tend to be much closer to the distribution of the true placebo effects (note the similarity between the blue and black densities).

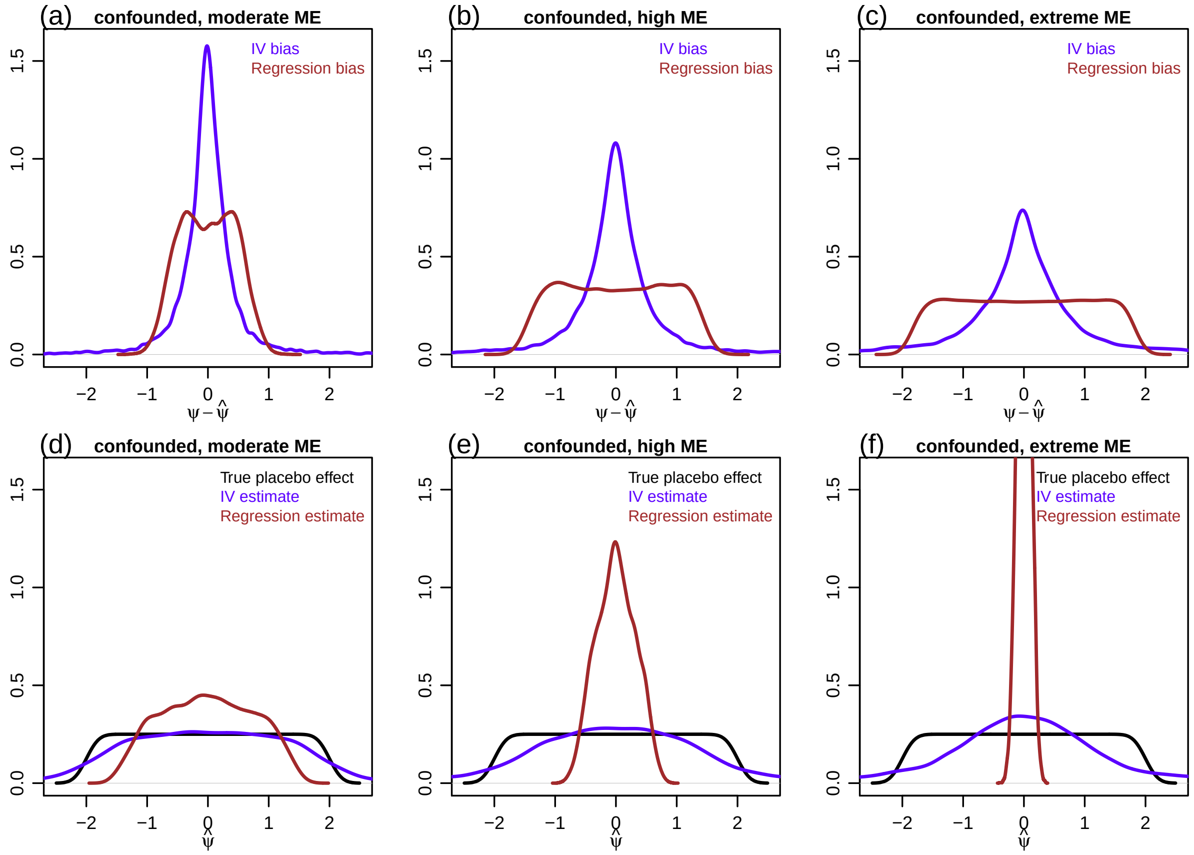

Figure 4. Empirical bias of the placebo effect tests under the influence of measurement error and in the presence of confounding. Panels a, b, and c show density estimates of the placebo effect bias, $\psi - \hat{\psi}$, for the IV (blue) and regression (brown) estimators, for the data sets simulated under the influence of moderate, high, and extreme amounts of measurement error of the emotion level variable, but in the presence of confounding. The panels clearly show that the IV estimator (blue) produces considerably less biased placebo effect estimates than the regression estimator (brown), and that the amount of bias tends to increase with increasing amounts of measurement error (as one would expect). Panels d, e, and f present the distributions of the placebo effects estimates generated by the IV (blue) and regression (brown) estimators along with the distributions of the true placebo effects (black) used to simulate the synthetic data. These panels show that the placebo effect estimates generated by the regression approach tend to get more concentrated around zero, as the amount of measurement error increases, whereas the estimates generated by the IV estimator tend to be closer to the distribution of the true placebo effects.

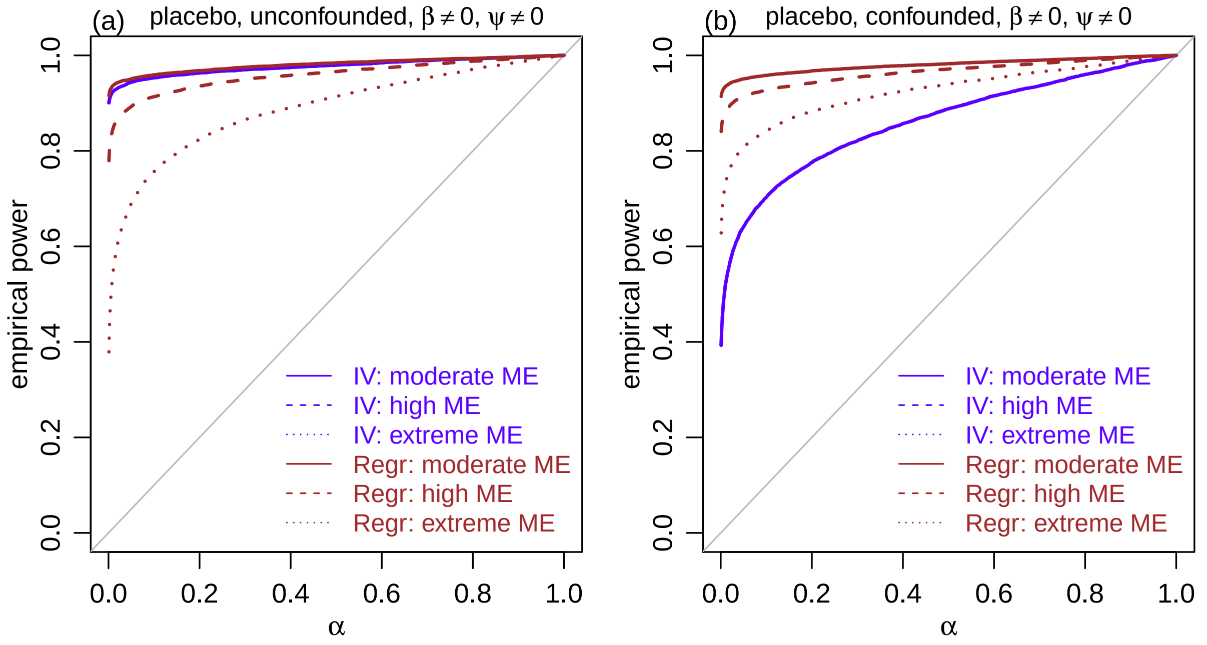

Figure 5. Empirical power of the placebo effect tests under the influence of measurement error. Panel a shows that in the absence of confounding, the IV estimators tended to show high empirical power (independent of the amount of ME used in the simulations), whereas the power of the regression approach tended to decrease with increasing amounts of ME. Panel b, on the other hand, show that the regression estimator tended to be more powered than the IV one in the presence of confounding. It also shows that the power of the regression approach tended to decrease with increasing amounts of ME, whereas the power curves of the IV approach were the same, independent of the amount of measurement error.

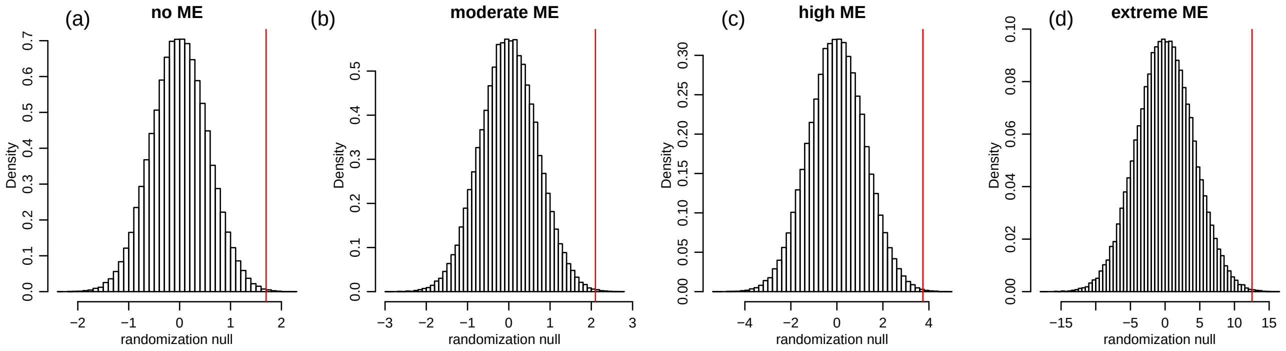

Figure 6. The randomization test for the placebo effect is robust to measurement error in the emotion level variable. Panels a, b, c and d present the randomization null distributions for the IV estimator of the placebo effect, under the "no ME", "moderate ME", "high ME, and "extreme ME" settings, respectively. The 4 null distributions were generated using the same random permutations of the response data. In this particular example, the true placebo effect used to simulate the data was $\psi = 1$, and the respective estimated effects were 1.698, 2.099, 3.737, and 12.527. The respective estimated covariances between the instrument and the emotion levels were 0.165, 0.134, 0.075, and 0.022. In spite of the increasing spreads of the null distributions (due to the decreasing covariances between the instrument and emotion level), in all 4 cases we have that exactly 95 out of the 100,000 permutations of the response data, lead to statistics equal or larger than the respective estimates in the original data (shown by the red lines). In all 4 cases we also observed that exactly 105 permutations of the response data, lead to statistics equal or less than the negative of the observed estimates. Therefore, the two-tailed p-values derived from the 4 randomization tests are identical and equal to 0.002.

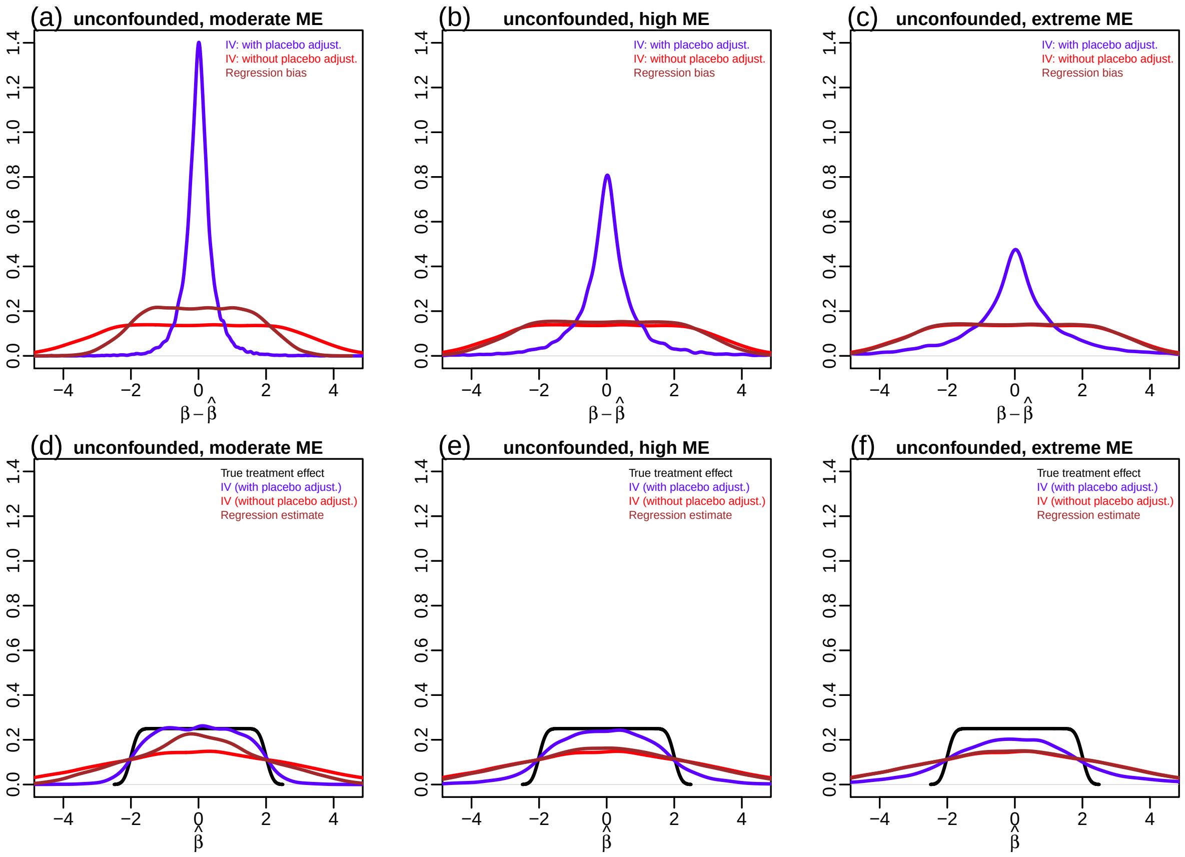

Figure 7. Empirical bias of the treatment effect tests under the influence of measurement error and in the absence of confounding. Panels a, b, and c show density estimates of the treatment effect bias, $\beta - \hat{\beta}$, for the regression (brown), placebo adjusted IV (blue) and unadjusted IV (red) estimators, for the data sets simulated under the influence of moderate, high, and extreme amounts of measurement error of the emotion level variable, but in the absence of confounding. The panels clearly show that the placebo adjusted IV estimator (blue) produces considerably less biased estimates than the regression (brown) and unadjusted IV (red) estimators, and that the amount of bias for the regression and placebo adjusted estimators tended to increase with increasing amounts of measurement error (note, however, that the bias is constant for the unadjusted IV estimator since it does not depend on the emotion level, M, and, hence, is not influence by measurement error on M). Panels d, e, and f present the distributions of the treatment effects estimates generated by the regression (brown), placebo adjusted IV (blue) and unadjusted IV (red) estimators along with the distributions of the true placebo effects (black) used to simulate the synthetic data. Note that the regression estimates (brown density) tend to approximate the unadjusted IV estimates (red density) as the amount of ME increases. The likely reason is that the regression estimator tends to automatically down play the contribution of $\tilde{M}$ to the response Y in the presence of extreme ME. In other words, $\hat{\psi}_R$ tends to be close to 0 (panel f of Figure 3), during the estimation of the parameters of the linear model $Y = \mu_Y + \beta X + \psi \tilde{M} + \varepsilon_Y$, so that the treatment effect estimate produced by the regression of Y on both X and M tends to be similar to the estimate produced by regression Y on X alone (whose value tends to be similar to the estimate generated by the unadjusted IV estimator, in the absence of confounding).

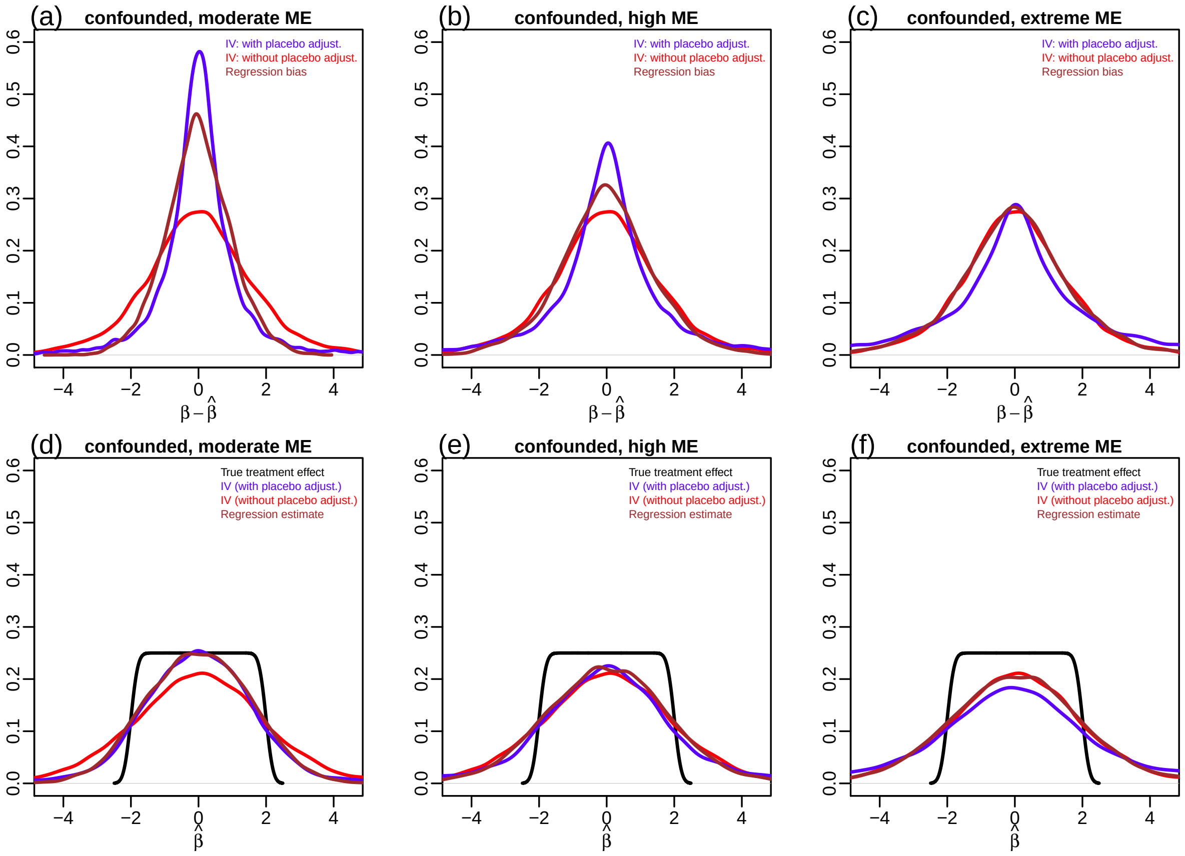

Figure 8. Empirical bias of the treatment effect tests under the influence of measurement error and in the presence of confounding. Panels a, b, and c show density estimates of the treatment effect bias, $\beta - \hat{\beta}$, for the regression (brown), placebo adjusted IV (blue) and unadjusted IV (red) estimators, for the data sets simulated under the influence of moderate, high, and extreme amounts of measurement error of the emotion level variable, but in the presence of confounding. Panel a shows that the placebo adjusted IV estimator (blue) produces less biased estimates than the regression (brown) and unadjusted IV (red) estimators in the presence of moderate amounts of measurement error. Panel b shows that the bias density of the placebo adjusted IV estimator is slightly more peaked around 0, but also shows heavier tails than the densities of the unadjusted IV (red) and regression (brown) estimators. Panel c, however, clearly shows that the placebo adjusted IV estimator can be more biased than the other estimators in the presence of extreme amounts of measurement error (note how the blue density puts less mass around 0, and have considerably heavier tails than the red and brown densities). Panels d, e, and f present the distributions of the treatment effects estimates generated by the regression (brown), placebo adjusted IV (blue) and unadjusted IV (red) estimators along with the distributions of the true placebo effects (black) used to simulate the synthetic data. These results illustrate that the presence of both confounding and measurement error makes the statistical inference considerably more challenging, specially in the presence of extreme amounts of measurement error.

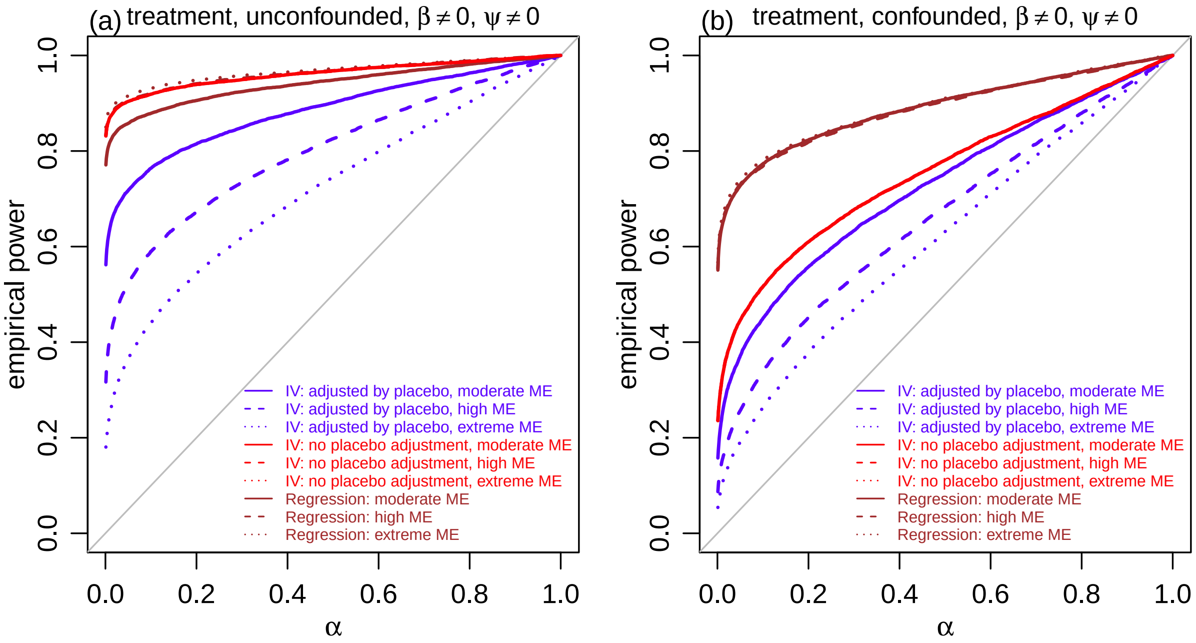

Figure 9. **Empirical power of the treatment effect tests under the influence of measurement error.** Panels a and b report the empirical power for the regression (brown), placebo adjusted IV (blue) and unadjusted IV (red) estimators, for the data sets simulated under the influence of moderate, high, and extreme amounts of measurement error of the emotion level variable, in the absence and in the presence of confounding, respectively. In both panels the regression and unadjusted IV estimators tended to be higher powered than the placebo adjusted IV estimator (whose power tended to decrease with increasing amounts of ME).

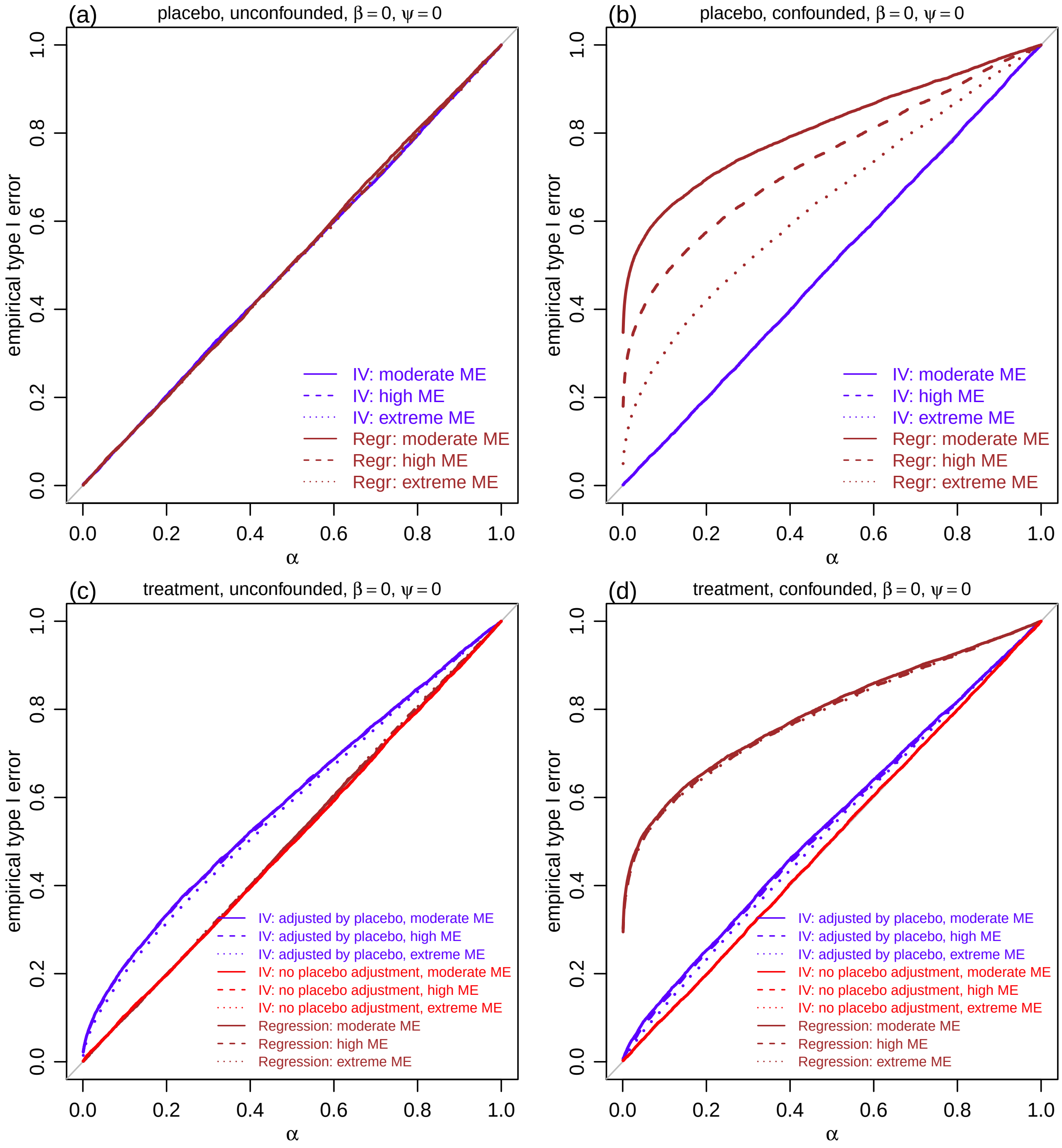

Figure 10. Empirical type I error rates of the placebo and treatment effect tests under the influence of measurement error. Panels a and b present the results for the placebo effect tests in the unconfounded and confounded cases, respectively, and show that the error rates of the IV approach (blue) are still controlled at the exact nominal levels in the presence of measurement error, whereas the regression approach (brown) still shows inflated error rates in the presence of confounding. Panels c and d present the respective results for the treatment effect tests. Both panels show well controlled error rates for the unadjusted IV approach (red), but slightly inflated error rates for the placebo adjusted IV approach (blue). The regression approach (brown), on the other hand, shows well controlled error rates in the absence of confounding (panel c) but inflated errors in the presence of confounding (panel d). Results based on 6 additional simulation experiments, each one encompassing 10,000 distinct data sets.
